# Novel Pacemaker Relocation Method for Radiotherapy Against Overlapping Lung Cancer

**DOI:** 10.7759/cureus.74343

**Published:** 2024-11-24

**Authors:** Atsuo Mori, Koji Funaishi, Hirofumi Haida, Kenya Nishizawa, Tatsuji Yoshimoto, Noritaka Shimizu, Tohru Kuribayashi

**Affiliations:** 1 Cardiovascular Surgery, Kawasaki Municipal Hospital, Kawasaki, JPN; 2 Cardiology, Kawasaki Municipal Hospital, Kawasaki, JPN; 3 Cardiology, Kawasaki Municipal Ida Hospital, Kawasaki, JPN; 4 Radiology, Kawasaki Municipal Hospital, Kawasaki, JPN

**Keywords:** lung cancer, pacemaker lead, pacemaker relocation, radiation therapy, stereotactic body radiotherapy

## Abstract

We experienced a case of a patient with a history of pacemaker implantation who was found to have lung cancer just behind the pacemaker. She was an 80-year-old woman with a history of valve replacement, pacemaker implantation, and sarcoidosis. Computed tomography showed a ground-glass opacity of 1.5 cm in diameter in her left lung just posterior to the pacemaker. Pathology revealed that the lesion was an adenocarcinoma of the lung. The patient required radiation therapy. The pacemaker needed to be relocated to avoid radiation exposure. An 85-cm-long lead was inserted from the contralateral vein. This lead was connected through a long subcutaneous tunnel to a new generator implanted in the abdomen. The patient received 60 Gray stereotactic body radiotherapy for lung cancer and the lesion regressed. Relocation of the pacemaker to the abdominal wall using a long transvenous lead may be a promising measure to allow radiotherapy when the pacemaker overlaps with a malignant tumor.

## Introduction

It is well known that cardiovascular implantable electronic devices, including pacemakers (PMs) and defibrillators, can be damaged by direct radiation exposure above a certain dose. According to the literature, the radiation tolerance of PMs is 2 to 5 Gray (Gy) [1,2]. Although rare, it is possible for a patient to develop a malignant tumor around the implanted PM. Even with adjacent PM, radiation therapy (RT) may be required to treat the cancer.

If there is some distance between the tumor and PM, the radiation dose affecting the PM can be reduced by planning the treatment. However, if the malignant tumor is inadvertently located directly behind the PM after implantation, it is impossible to avoid radiation, no matter how tactical the radiation planning could be. In such cases, surgery to relocate the PM is inevitable. Various methods of pacemaker implantation (PMI) have been reported in the past to deal with this situation, but they remain controversial [3-5].

In this paper, we present a new technique we developed in which a long lead is inserted from the contralateral side, and the PM is relocated to the abdomen when the left upper lobe lung cancer overlaps the PM.

## Case presentation

**Figure 1:**
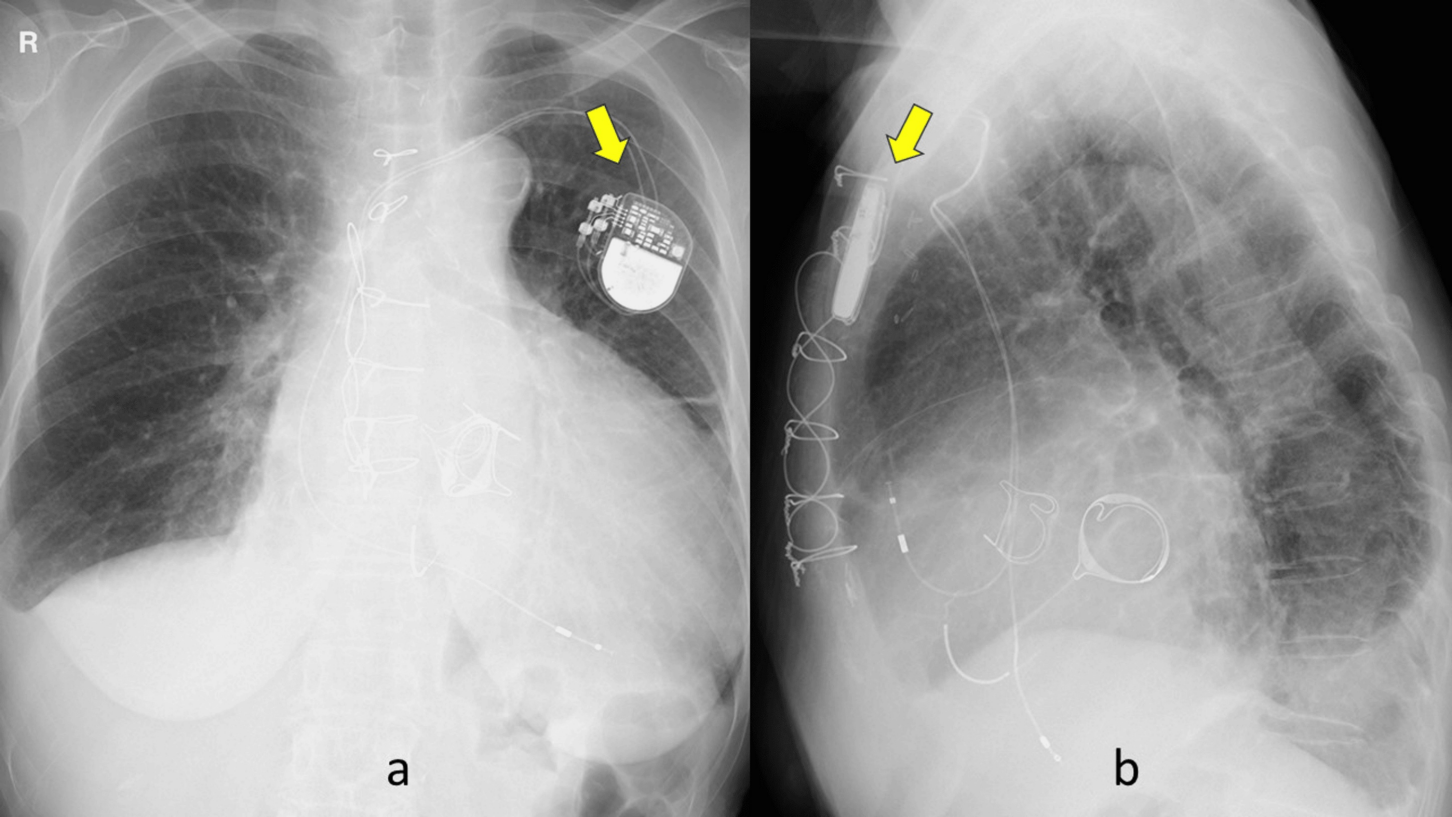
Chest X-ray films. Arrows show the implanted pacemaker. Ground glass opacity in the left upper lobe of the lung cannot be seen. (a) Frontal and (b) lateral.

The patient was an 80-year-old woman who had been attending our outpatient clinic for the treatment of sarcoidosis. She had undergone aortic valve replacement, mitral valve replacement, and tricuspid valve annuloplasty at another hospital in 2019. At that time, an intraoperative myocardial biopsy revealed cardiac sarcoidosis. After the surgery, the patient developed severe bradycardia due to a complete atrioventricular block and underwent dual-chamber PMI (Figure 1).

**Figure 2:**
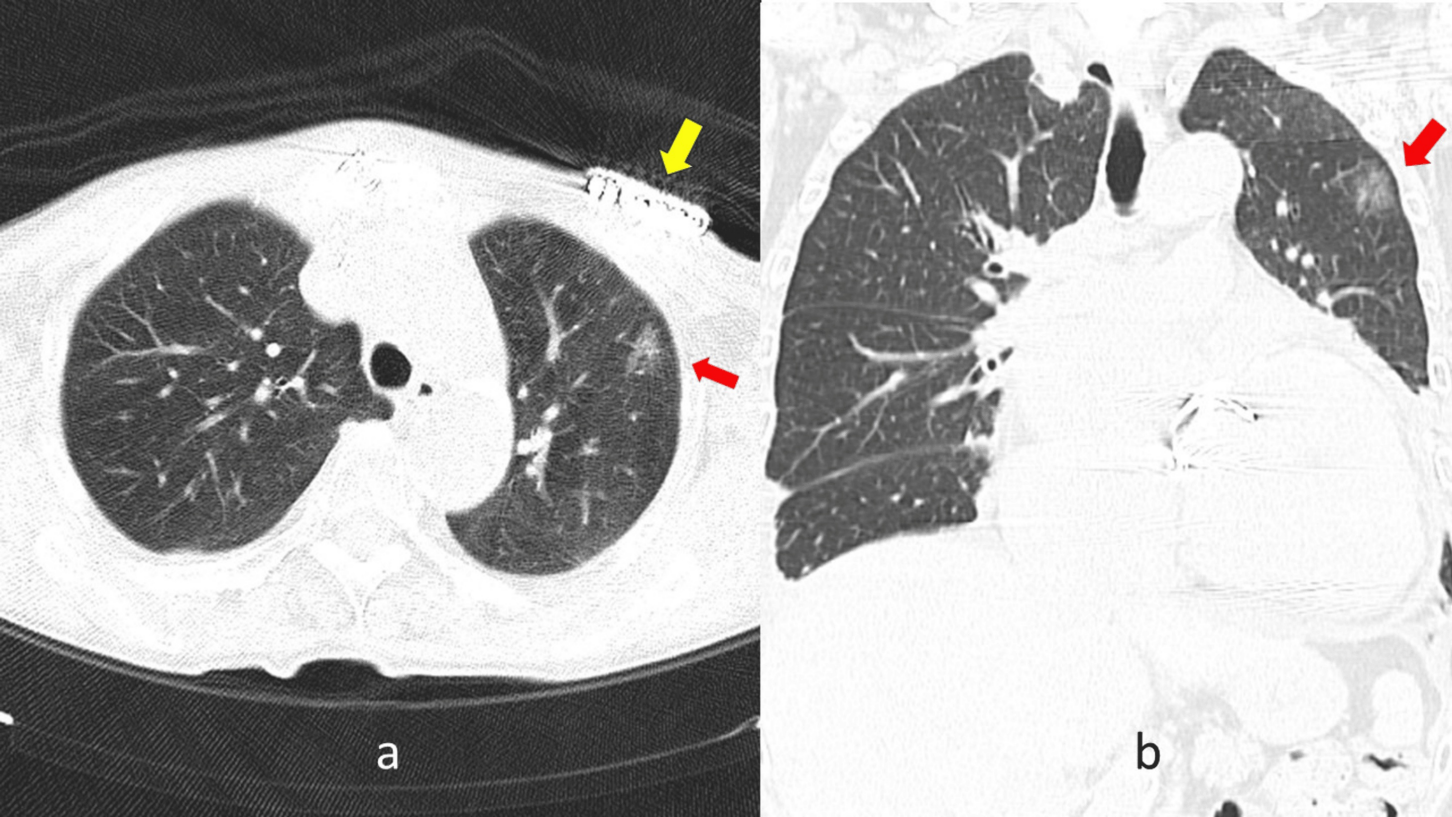
CT scan films before operation. Red arrows show ground glass opacity just behind the pacemaker. The yellow arrow shows the implanted pacemaker. (a) Cross-sectional view and (b) frontal view.

In 2020, during an outpatient visit to our internal medicine department, a follow-up computed tomography (CT) scan for pulmonary sarcoidosis revealed ground glass opacity (GGO) in the left upper lung field. The lesion was 1.5 cm in size, and it was necessary to differentiate between lung cancer and pulmonary sarcoidosis (Figure 2).

There was no obvious abnormal shadow on the chest X-ray film (CXP) at that time (Figure 1).

One of the hot topics in lung cancer is GGO, which is not visible on chest X-ray but can be detected on chest CT. Of all GGOs, 80-90% are lung cancers, but 10-20% are nonmalignant and resolve spontaneously [6]. If GGO does not resolve over time, early-stage lung adenocarcinoma is suspected. Therefore, a thorough evaluation including bronchoscopy and sputum cytology is necessary to make a definitive diagnosis.

**Figure 3:**
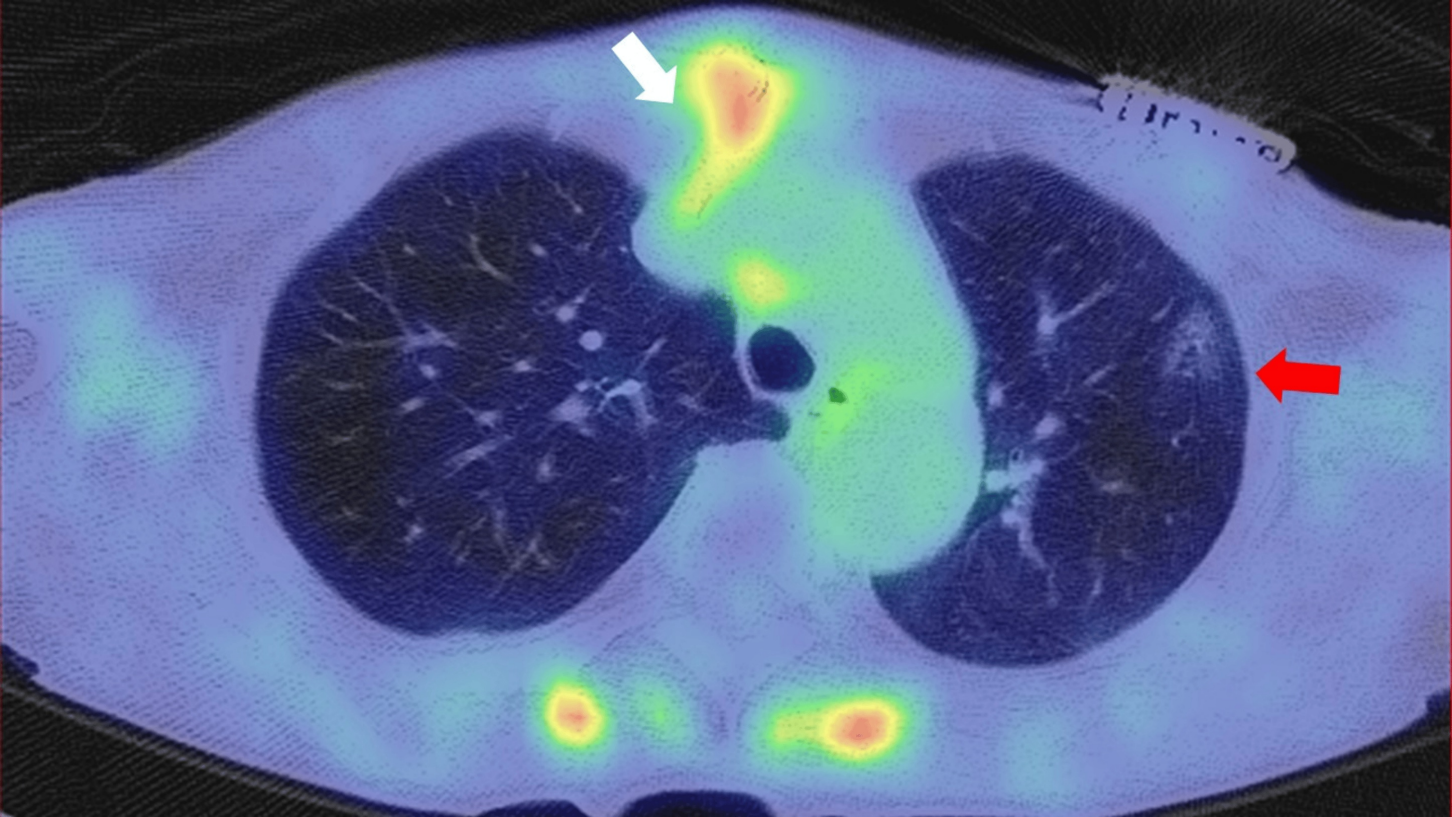
Positron emission tomography. Ground glass opacity immediately behind the pacemaker without uptake (red arrow). Sternum and aortic arch with uptake (white arrow).

Positron emission tomography (PET) was performed and showed GGO in the left upper lung at the same site as seen on CT. However, PET showed no uptake of radioisotope or 18F-fluorodeoxyglucose (18F-FDG) at the site. There was increased uptake in the aortic arch and sternal lymph nodes, which was considered to be caused by inflammation following cardiac surgery (Figure 3).

A CT-guided biopsy was performed for a differential diagnosis of pulmonary sarcoidosis or malignancy. Pathology revealed atypical cells and a diagnosis of pulmonary adenocarcinoma was made. There were no metastases to distant organs, including the brain. The stage of lung cancer was T1aN0M0 and the stage classification was stage I.

The patient had poor cardiac function with an ejection fraction <30% due to a sarcoid heart. She had also undergone valve replacement. Therefore, RT was considered more appropriate than pulmonary resection. The patient was informed and consented to the treatment. However, the problem was that the tumor and the PM completely overlapped in the radiation field. Simple contralateral implantation of the PM was considered inappropriate because of the exposure of the PM to lateral radiation.

We then considered the possibility of using an epicardial lead to deliver the PM to the abdominal wall. We had seen a similar situation prior to this case in which the PM was repositioned to the abdomen using an epicardial lead and RT was successfully completed [6]. However, since the present case was postoperative for valvular heart disease and a median sternotomy had already been performed, we decided that this method was not appropriate for the present case.

**Figure 4:**
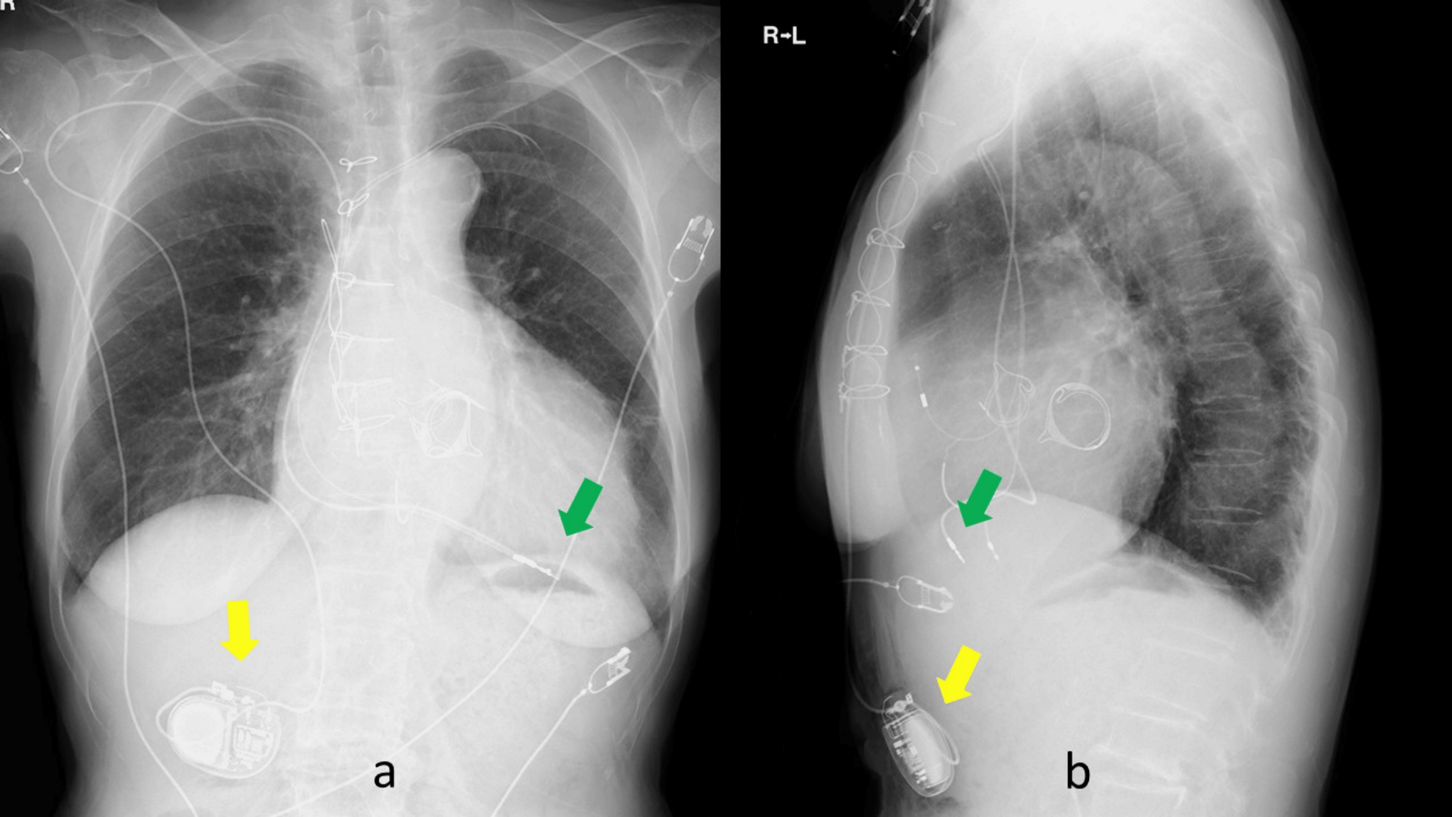
Chest X-ray films. Yellow arrows indicate the relocated pacemaker in the abdomen. Green arrows indicate the tip of the pacing lead at the right ventricle. (a) Frontal and (b) lateral.

Therefore, we developed a novel method using a long lead. The long lead was introduced from the contralateral side and placed with the tip in the right ventricle. The lead connector was then passed subcutaneously and connected to the PM in the abdominal wall. To our knowledge, this is the first case report using this technique (Figure 4).

**Figure 5:**
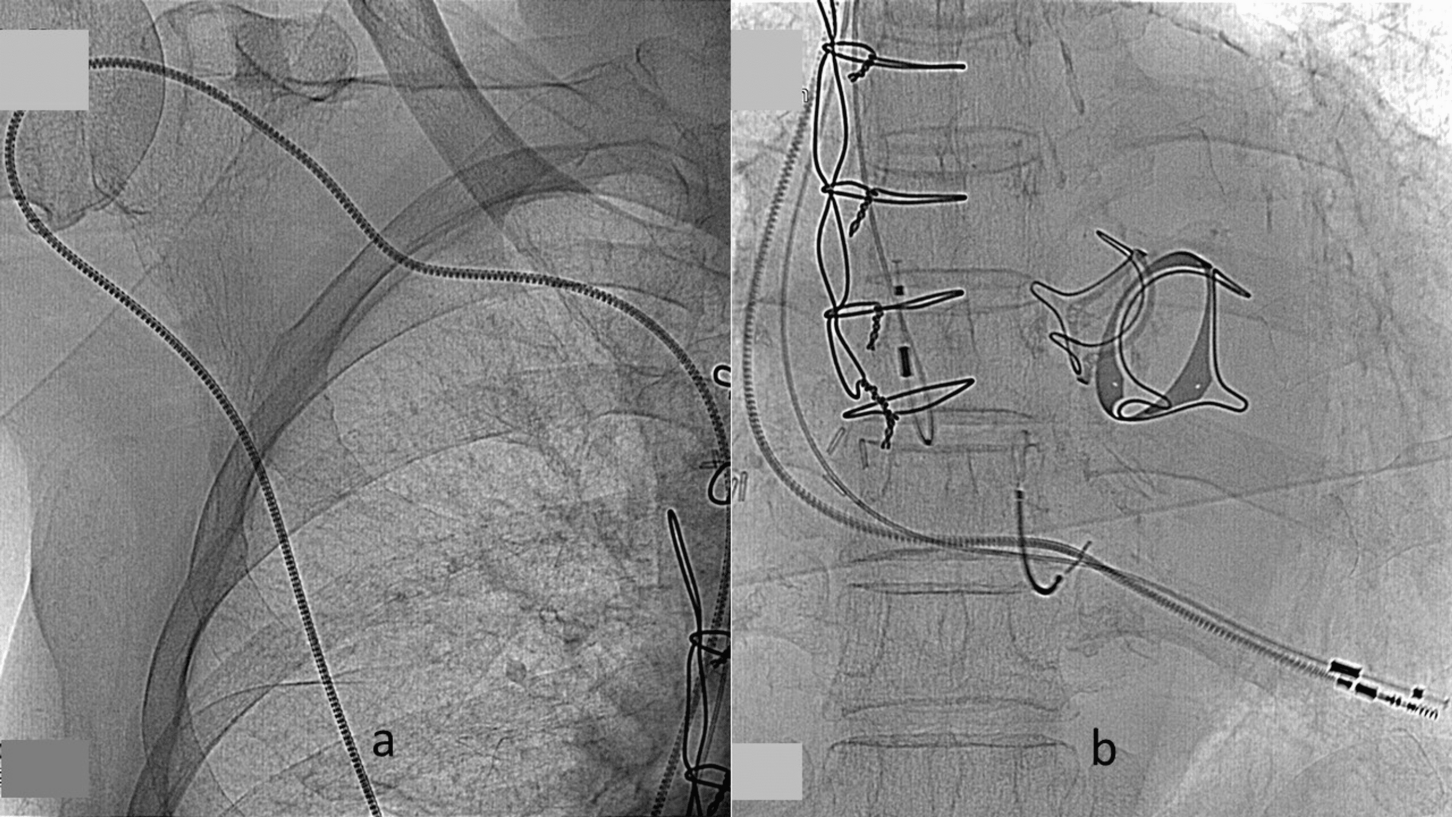
Intraoperative fluoroscopy. (a) An 85-cm-long lead was introduced via the right axillary vein. (b) The screwed tip of the pacing lead was fixed to the right ventricle.

The procedure was as follows. Under local anesthesia, a pacing lead was inserted through the right axillary vein, the lead was guided under fluoroscopic guidance, and its tip was implanted into the right ventricle. The lead was 85 cm long (Medtronic, Dublin, Ireland) originally designed for very large patients. The tip was fixed to the right ventricular apex with a coaxial rotation screw (Figure 5).

**Figure 6:**
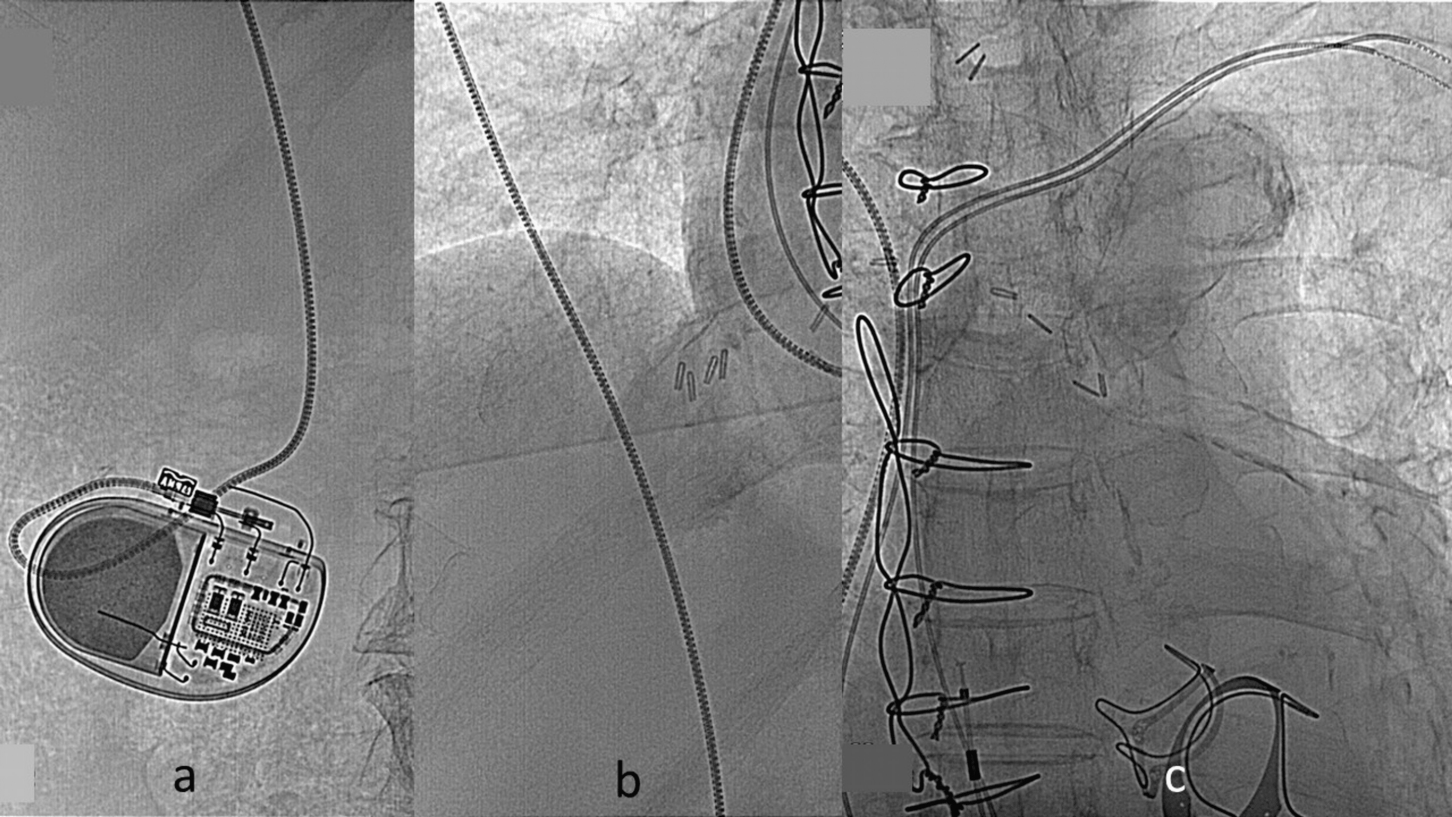
Intraoperative fluoroscopy. (a) Pacemaker translocated in the abdomen. (b) An 85-cm-long lead was passed through the subcutaneous tissue. (c) The lead ends were cut off.

In situ measured wave height, resistance, and pacing threshold were satisfactory. A combination of subcutaneous local anesthesia and intravenous anesthesia with dexmedetomidine hydrochloride (DH) was used, and a plastic tube for intravenous injection was placed subcutaneously through a subcutaneous delivery device. The PM lead was passed through a layer of subcutaneous tissue to the abdominal wall. The PM body was connected to the lead and implanted in a pocket under the rectus abdominis fascia. The previously implanted pacemaker was removed. The atrial and ventricular lead ends were cut off and covered with a plastic sheath (Figure 6).

The postoperative course was uneventful. RT for lung cancer was performed two weeks after discharge. Outpatient, the patient received 60 Gy/5 fractions stereotactic body radiotherapy (STBR). PM checks performed before and after RT showed no problems with threshold, resistance, or sensitivity. There were no adverse events during this period, including radiation-induced pacing failure or return to default settings.

**Figure 7:**
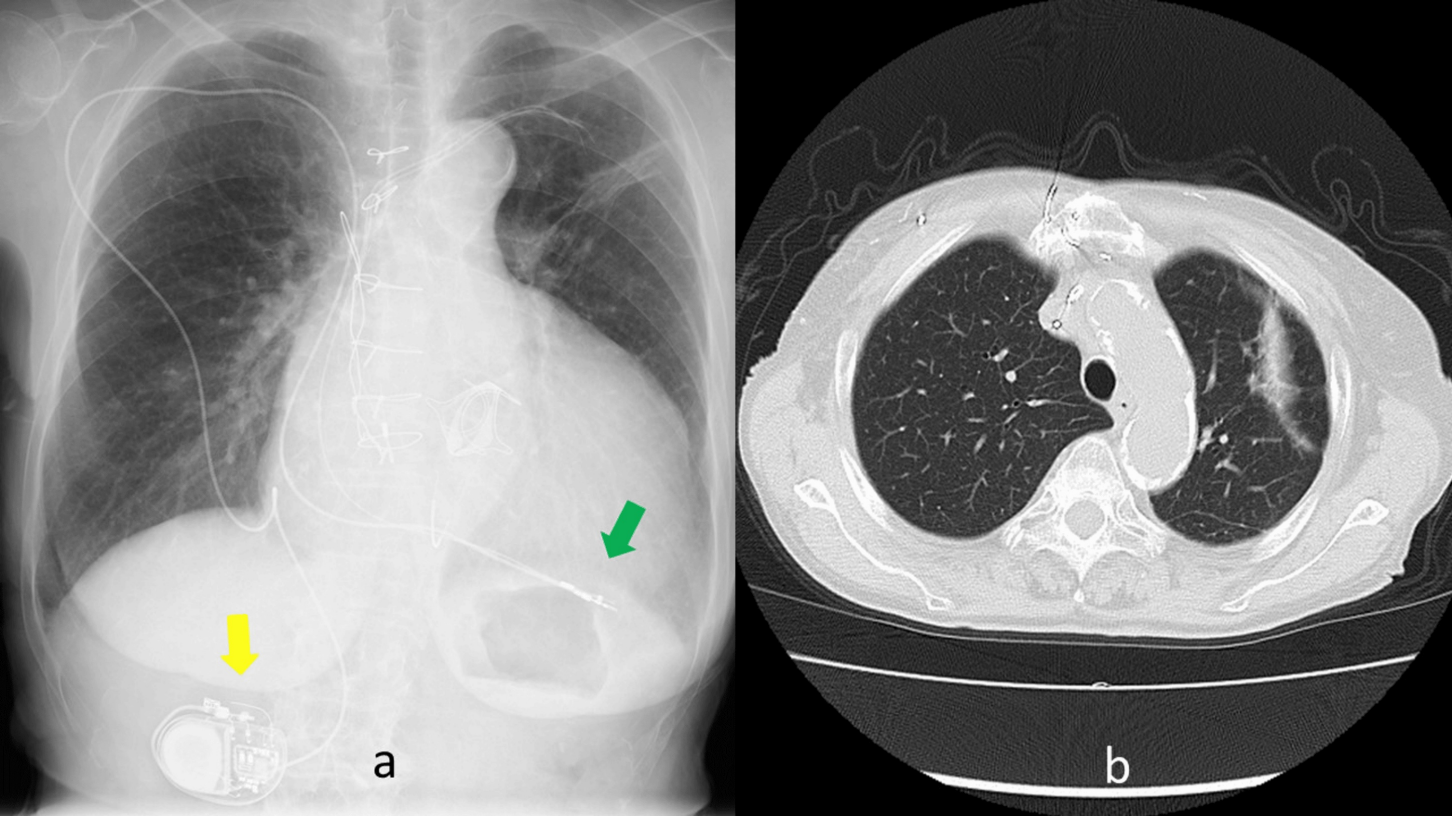
Chest X-ray film and CT scan. (a) The yellow arrow indicates the translocated pacemaker. The green arrow indicates the tip of the pacing lead. Consolidation can be seen in the left upper lobe. (b) CT at four years and two months after radiation therapy. Only scar exists.

Neither chemotherapy with anti-cancer drugs nor immunotherapy with immune checkpoint blockade drugs were used. It has been four years and 10 months since radiation. PM is normal with 100% pacing and no threshold or other problems. No syncope, epilepsy, altered consciousness, bradycardia, or tachycardia have been observed during the course of the disease. The most recent CXP showed only a slight cordlike appearance of the irradiated area (Figure 7). CT showed complete resolution of the GGO lesion (Figure 7).

There was still evidence of local inflammation, but with time, it became a scar. RT seemed to be very effective against the lung cancer lesion in this case.

Regarding cardiac function, echocardiography revealed severe tricuspid regurgitation. Her ejection fraction was less than 20% and she also had symptoms of heart failure. However, cardiac sarcoidosis was treated with oral steroids, and her EF improved to about 30%. In 2024, she is now able to live her daily life without problems, although she has some dyspnea on exertion.

## Discussion

In recent years, the prevalence of lung cancer has increased, as has the number of PMs and other devices implanted [7,8]. RT is a treatment in which X-rays or neutron beams are delivered to the affected area to damage the DNA of cancer cells. However, it can also damage PMs and implantable defibrillators, which are made of delicate and complex semiconductors [1,2]. For PMs at a distance from the tumor, irradiation can be avoided by shielding, such as mantle irradiation, or by adjusting the size and direction of the irradiation field. However, if the lung cancer is located immediately behind or in the immediate vicinity of the PM, radiation exposure to the PM associated with RT cannot be avoided.

While there are discrepancies among PM manufacturers, the accepted radiation dose for PMs is generally considered to be within the range of 2 to 5 Gy [1,2]. What adverse events would occur if therapeutic radiation exposure to PMs exceeded this amount? According to the literature to date, the effects of radiation exposure on electronic devices such as PMs fall into two categories. One is transient, whereby memory and other settings may be altered [9]. The other is irreversible damage, whereby the electronic circuits themselves are materially destroyed and cannot be normalized even after interrogation. Furthermore, radiation exposure may render it impossible to interrogate the PM [10].

The means to deal with this situation would primarily depend on the distance between the tumor and the PM. First, if the irradiation site and the implanted PM are far enough apart, normal irradiation can be used without problems. Second, if the patient has a certain degree of intrinsic rate and the tumor and PM are relatively close but not overlapping, the radiation dose can be reduced by special arrangements in the radiation plan. However, even in this case, backup external pacing may be necessary in patients with a 100% pacing rate and no intrinsic rhythm, or with a low intrinsic rate and a high pacing threshold. If there is no escape beat at all and the pacing rate is 100%, radiation-induced pacing failure can have serious consequences for the patient. Finally, what if the tumor and the PM are completely overlapping, as in this case of lung cancer? If the lung cancer is located directly behind the PMI site, it is essential to move the PM if radiation is to be used as a therapy [1].

What methods of PM translocation have been documented in the literature? The most common method is to transfer the body to the contralateral side. This method can be performed under local anesthesia, but it has the disadvantage of exposing the new PM to radiation from the lateral side. Furthermore, in the event of the emergence of a new lesion in the lung on the side where the PM was relocated, the necessity for a subsequent relocation may arise [3].

An alternative method is to transfer the side or PM to the abdomen by extending it with a connecting lead, which is not a commonly employed technique. In this instance, the end of an existing lead would be employed for an unintended purpose [4].

An alternative approach is the utilization of an epicardial lead, as we have previously reported. This method entails the placement of the PM in the abdomen, thereby obviating the issue of lateral radiation and allowing for the RT plan to be followed with minimal restrictions [5]. The procedure is somewhat invasive, necessitating general anesthesia with tracheal intubation and a partial sternotomy. Indeed, in the event of postoperative adhesion of the pericardium, the procedure becomes challenging due to the necessity of dissecting the adhesion.

In the present case, the patient had a history of prosthetic valve replacement, median sternotomy, and adhesions between the heart and pericardium. Additionally, she exhibited evidence of sarcoidosis and a reduced ejection fraction on echocardiography. We concluded that the aforementioned procedure with an epicardial lead would be unduly invasive and unsuitable for this patient.

In light of the aforementioned techniques, we have devised this method as a more sophisticated and advanced technique. As the PM is relocated to the abdomen, there are no constraints on radiation from any direction, and further radiation is readily feasible even in the event of contralateral lung lesions or enlarged mediastinal lymph nodes. The procedure can be performed under local anesthesia, technically. The procedure is not affected by adhesions resulting from previous cardiac surgery.

This method permits the evacuation of the pacemaker body at a sufficient distance from the lesion. Consequently, it is advantageous that the radiation therapist is not required to devise a highly complex and bespoke radiation plan to avoid irradiating the pacemaker and can instead perform radiation therapy in a manner that is largely consistent with standard practice.

One disadvantage of this technique is that it increases the number of pacing leads passing through the tricuspid valve. Tricuspid valve regurgitation may result in worsening of the condition, leading to enlargement of the right atrium and subsequent development of right heart failure. Due to the length of the pacing lead (85 cm), it may prove more challenging than usual to guide the lead to the optimal right ventricular location. This procedure requires a higher level of experience and technical proficiency of the operator. Additionally, the lead must be passed through a lengthy subcutaneous tunnel within the chest wall, which is a relatively uncommon occurrence. The utilization of supplementary intravenous anesthesia, such as DH, during the subcutaneous passage of a single pacing lead through the chest wall may assist in mitigating patient discomfort.

STBR uses a very small radiation field, which is the reason it reduces radiation damage to normal areas without spreading to surrounding areas. This means that STBR allows re-irradiation even in cases of recurrence. The ability to deliver additional radiation even if the lung cancer recurs ipsilaterally or contralaterally is a major advantage [11]. This is why we believe that the PM transfer method presented in this paper is particularly compatible with STBR.

## Conclusions

We have developed a new method of pacemaker relocation using a long lead for radiotherapy of GGO generated behind an already implanted pacemaker. The patient who actually adapted this method completed the radiotherapy without any problems and is alive four years and 10 months later. We believe that this is an effective and beneficial surgical technique for treating lung cancer behind a pacemaker.
